# Supplementary material for: Foliar Application of Sodium Nitroprusside Boosts Solanum lycopersicum L. Tolerance to Glyphosate by Preventing Redox Disorders and Stimulating Herbicide Detoxification Pathways
Source: Plants (Basel). 2021 Sep 9;10(9):1862. doi: 10.3390/plants10091862 (PMC8466062; doi:10.3390/plants10091862)
Supplement: Supplementary file 1 [file plants-10-01862-s001.zip › plants-1347957-supplementary.pdf]

Supplementary Material

Table S1. Detailed ANOVA results for all evaluated parameters in roots of *Solanum lycopersicum* L. cv. Micro-Tom grown for 28 days in OECD soil contaminated by GLY (10 mg kg-1) and/or foliar treated with SNP (200 μM). Parameters where significant differences ($p \leq 0.05$) were recorded are highlighted at bold.

Parameter	ANOVA
**Root length**	**F (3, 10) = 19.11; *p* < 0.01**
**Fresh biomass**	**F (3, 9) = 64.36; *p* < 0.01**
**NR**	F (3, 8) = 3.013; *p* > 0.05
**Total protein**	**F (3, 10) = 19.21; *p* < 0.01**
**LP**	**F (3, 9) = 9.339; *p* < 0.01**
**$O_2^{\cdot-}$**	**F (3, 16) = 12.03; *p* < 0.01**
**H_2O_2**	**F (3, 8) = 12.29; *p* < 0.01**
**Proline**	**F (3, 12) = 14.17; *p* < 0.01**
**GSH**	**F (3, 8) = 46.08; *p* < 0.01**
**Total AsA**	**F (3, 8) = 7.842; *p* < 0.01**
**AsA/DHA**	**F (3, 13) = 5.991; *p* < 0.01**
**TAC**	**F (3, 8) = 4.792; *p* < 0.05**
**TPC**	F (3, 8) = 0.3788; *p* > 0.05
**SOD**	**F (3, 9) = 10.36; *p* < 0.01**
**CAT**	**F (3, 9) = 13.45; *p* < 0.01**
**APX**	**F (3, 8) = 23.07; *p* < 0.01**
**GST**	**F (3, 9) = 50.51; *p* < 0.01**

Table S2. Detailed ANOVA results for all evaluated parameters in shoots of *Solanum lycopersicum* L. cv. Micro-Tom grown for 28 days in OECD soil contaminated by GLY (10 mg kg-1) and/or foliar treated with SNP (200 µM). Parameters where significant differences ($p \leq 0.05$) were recorded are highlighted at bold.

Parameter	ANOVA
**Fresh biomass**	**F (3, 10) = 13.74; *p* < 0.01**
**NR**	**F (3, 8) = 7.339; *p* < 0.05**
**Total protein**	**F (3, 27) = 3.902; *p* < 0.05**
**LP**	**F (3, 8) = 14.02; *p* < 0.01**
**$O_2^{\cdot-}$**	**F (3, 17) = 20.23; *p* < 0.01**
**H_2O_2**	F (3, 10) = 1.127; *p* > 0.05
**Proline**	**F (3, 9) = 28.02; *p* < 0.01**
**GSH**	**F (3, 15) = 14.83; *p* < 0.01**
**Total AsA**	**F (3, 9) = 7.387; *p* < 0.01**
**AsA/DHA**	**F (3, 9) = 5.532; *p* < 0.05**
**TAC**	F (3, 8) = 1.445; *p* > 0.05
**TPC**	**F (3, 5) = 49.63; *p* < 0.01**
**SOD**	F (3, 9) = 2.874; *p* > 0.05
**CAT**	**F (3, 7) = 27.41; *p* < 0.01**
**APX**	**F (3, 9) = 7.474; *p* < 0.01**
**GST**	**F (3, 9) = 6.360; *p* < 0.05**

Table S3. Detailed ANOVA results for productivity-related parameters of *Solanum lycopersicum* L. cv. Micro-Tom grown for 28 days in OECD soil contaminated by GLY (10 mg kg-1) and/or foliar treated with SNP (200 µM). Parameters where significant differences ($p \leq 0.05$) were recorded are highlighted at bold.

Parameter	ANOVA
**Number of flowers**	**F (3, 10) = 4.444; *p* < 0.05**
**Number of fruits**	**F (3, 14) = 4.370; *p* < 0.05**
**Fruit fresh biomass**	F (3, 16) = 0.571; *p* > 0.05
